# Repurposing Statins for Renal Protection: Is It a Class Effect?

**DOI:** 10.1111/cts.12521

**Published:** 2017-11-15

**Authors:** Stephen J. McWilliam, Daniel J. Antoine, Munir Pirmohamed

**Affiliations:** ^1^ Department of Women's and Children's Health University of Liverpool Merseyside UK; ^2^ Department of Molecular and Clinical Pharmacology, and MRC Centre for Drug Safety Science University of Liverpool Liverpool Merseyside UK

## INTRODUCTION

There has been a great deal of excitement regarding the potential benefits of statins beyond their lipid‐lowering effect, and repurposing them for other indications. In this commentary, we evaluate the role of statins in protecting the kidneys, with a focus on three areas: cardiac surgery, contrast‐induced nephropathy, and aminoglycoside‐induced nephrotoxicity.

## COMMENTARY

Statins inhibit the activity of 3‐hydroxy‐3‐methylglutaryl CoA (HMG‐CoA) reductase in order to reduce cholesterol synthesis, leading to lower circulating concentrations of total cholesterol and low‐density lipoprotein. In adults, statins have proven efficacy in reducing morbidity and mortality, and improving clinical outcomes, in those at risk of vascular disease. Beyond their lipid‐lowering effect, statins have been reported to have a number of additional beneficial effects, in particular antiinflammatory and antioxidant effects.^1^ This has resulted in their consideration, among other indications, as potential renal protective agents

## POSTOPERATIVE ACUTE KIDNEY INJURY (AKI) FOLLOWING CARDIAC SURGERY

Cardiac surgery is associated with postoperative AKI in up to 30% of patients. Etiologically, this is thought to be due to a combination of ischemia and inflammation. Observational studies in patients undergoing cardiac surgery who were already on statins suggested a reduced incidence of AKI, when compared with those not on statins.^2^ However, a recent meta‐analysis of randomized clinical trials failed to show that preoperative statins reduced the incidence of postoperative AKI or requirement for renal replacement therapy.^3^ Various statins were used in these trials (including rosuvastatin, atorvastatin, and simvastatin), but the meta‐analysis grouped all statins together. This may be a limitation of the meta‐analysis, as there may be differences in the effects of different statins (and doses). Furthermore, the analysis was very dependent on the results of one large negative trial using rosuvastatin.

## CONTRAST‐INDUCED NEPHROPATHY

Systemic injection of contrast media plays a vital role in imaging for both diagnostic and therapeutic interventions. However, contrast media can cause acute kidney injury, with an incidence of less than 5% in low‐risk patients, increasing up to 50% in those with diabetes mellitus or chronic kidney disease. The mechanisms are complex and multifactorial, including a decrease in nitric oxide production, increase in endothelin production, inflammation, and the production of reactive oxygen species. These factors have been postulated to reduce renal perfusion and oxygenation, primarily damaging the tubular epithelium and renal vascular endothelium. Statins have been shown to maintain nitric oxide production, inhibit the production of reactive oxygen species, and have other beneficial effects on endothelial function.^1^ A recent meta‐analysis of 21 randomized controlled trials concluded that short‐term treatment with any statin (the trials used rosuvastatin, atorvastatin, or simvastatin) was associated with a significant reduction in contrast‐induced nephropathy (absolute risk reduction 43%).^4^ A subgroup analysis suggested that the evidence for a reduction in risk with atorvastatin and rosuvastatin was good, while it was of low quality with simvastatin. However, without further mechanistic insight and careful analysis of the dose–response relationship, it is not yet possible to conclude which statin is the most protective for this indication.

## AMINOGLYCOSIDE‐INDUCED NEPHROTOXICITY

Aminoglycoside antibiotics are generally used in clinical practice to provide Gram‐negative cover in the empirical treatment of suspected sepsis, or to provide targeted therapy, such as in the treatment of pulmonary exacerbations in children with cystic fibrosis (CF) colonized with *Pseudomonas aeruginosa*. However, the use of aminoglycosides is limited by their potential to cause nephrotoxicity.

**Figure 1:**
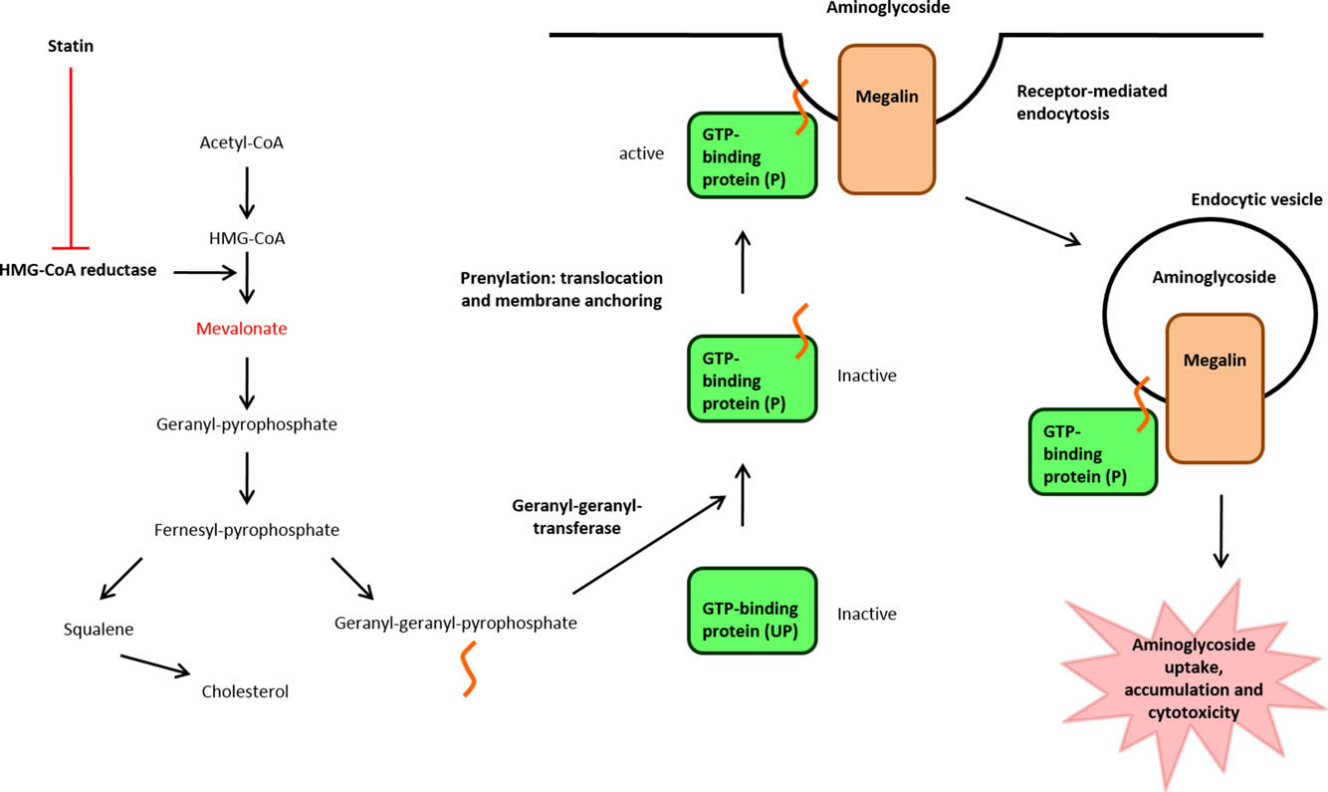
Mechanism of aminoglycoside‐induced cytotoxicity in proximal tubule epithelial cells, and proposed mechanism for inhibition by statins. Adapted from Biochemical Pharmacology, Antoine, D.J. *et al*. Statins inhibit aminoglycoside accumulation and cytotoxicity to renal proximal tubule cells, **79**, 647–654, ©2010, with permission from Elsevier.

Aminoglycoside‐induced nephrotoxicity is characterized by selective targeting of the proximal tubule epithelial cells within the renal cortex. Intracellular aminoglycoside results in apoptosis and necrosis of these cells via a variety of pathways (including mitochondrial dysfunction and the release of reactive oxygen species) (**Figure** 1).^5^ Aminoglycosides accumulate in proximal tubule cells through endocytosis via the multi‐ligand receptor, megalin. Consistent with this, in megalin knockout mice, there is no renal accumulation of aminoglycosides.^6^

Megalin‐mediated endocytosis is dependent on active guanosine‐5'‐triphosphate (GTP)‐binding proteins, which require posttranslational modification for trafficking to, and anchoring in, the cell membrane. Two isoprenoid intermediates, farnesyl pyrophosphate and geranylgeranyl pyrophosphate, play important roles in the prenylation of GTP‐binding proteins. These isoprenoid intermediates are derived from mevalonate, the downstream product of HMG‐CoA (**Figure** 1).^1^

*In vitro*, statins reduce cholesterol synthesis and increase unprenylated GTP‐binding proteins, leading to inhibition of megalin‐mediated endocytosis.^7^ In an opossum kidney cell model, statins (simvastatin, pravastatin, and rosuvastatin) were shown to inhibit cholesterol synthesis in a dose‐dependent manner.^8^ They also led to a dose‐dependent inhibition of gentamicin accumulation and cytotoxicity (measured by lactate dehydrogenase leakage), which were related to the degree of GTP‐binding protein unprenylation. Addition of mevalonate reversed the protective effect of statins, suggesting that inhibition of gentamicin‐induced cytotoxicity is directly related to the inhibition of HMG‐CoA and not to any off‐target effect of statins.

Our group has investigated this hypothesis further using an *in vivo* guinea pig model (unpublished data). This showed that gentamicin‐induced nephrotoxicity could be inhibited by rosuvastatin but not by simvastatin, providing evidence of a differential effect between the two statins, in contrast to our findings *in vitro*. This is likely to be due to the different pharmacokinetics of the two statins *in vivo*: rosuvastatin has greater renal elimination through proximal tubule secretion,^9^ while simvastatin is much more lipophilic and requires hepatic metabolism for elimination.

These findings informed the design of a phase IIa randomized controlled clinical trial of rosuvastatin for the prevention of aminoglycoside‐induced nephrotoxicity in children with CF (The PROteKT study; EudraCT 2014‐002387‐32, UKCRN ID 16993, ISRCTN26104255). In this multicenter trial, 50 children with CF receiving clinically indicated treatment with aminoglycosides were randomized equally to cotreatment with rosuvastatin or to current standard of care. A dose of 10 mg rosuvastatin was used in the trial based on allometric scaling from the guinea pig experiments. Recruitment to this trial is now complete, and results will be published in the near future. This phase IIa study, if positive, will be used to design a multicenter, phase III trial to evaluate the effect of rosuvastatin in preventing aminoglycoside‐induced kidney injury.

## CONCLUSION

This commentary has showcased the exciting area of drug repurposing in relation to the use of statins for renal protection, which is likely to have significant benefits for patients. To date, the evidence does not support a protective role for statins in the prevention of postcardiac surgery AKI. However, there is good evidence for a protective effect of statins in contrast‐induced nephropathy, although further work is required to determine the best statin and dose. Finally, results are awaited from a clinical trial to identify whether rosuvastatin may protect against aminoglycoside‐induced nephrotoxicity.

Statins all inhibit cholesterol production, but they vary in their potency with differing dose–response profiles. Furthermore, it is wrong to assume that any drug, including a statin, will have the same dose–response profile in a repurposed indication as it does in its original indication. Similarly, each statin is likely to differ in its pleiotropic effects. These effects are dependent on their individual chemical structure and pharmacology, including disposition. For instance, a hydrophilic statin, such as rosuvastatin, has greater renal excretion compared with lipophilic statins such as atorvastatin and simvastatin, suggesting it is likely to have a greater renal effect. Furthermore, rosuvastatin is secreted in the proximal tubules,^9^ which may explain its greater propensity to cause proteinuria,^10^ suggesting it is a more potent inhibitor of megalin‐mediated endocytosis, and therefore more likely to be renoprotective in aminoglycoside exposure. In contrast, where the renoprotective mechanism may be related to antioxidant or antiinflammatory effects, other statins may be more potent, potentially explaining the utility of atorvastatin for contrast‐induced nephropathy.

In conclusion, we would therefore suggest that identifying the correct statin and dose is crucial before taking a statin forward into large‐scale clinical trials for each new repurposed indication. This will require a combination of more extensive preclinical work in animal models and dose‐ranging experimental medicine studies in patients. “Preparation is the key to success” (Alexander Graham Bell).

## References

[1] Bonetti, P.O. , Lerman, L.O. , Napoli, C. & Lerman, A. Statin effects beyond lipid lowering—are they clinically relevant? Eur. Heart J. 24, 225–248 (2003). 12590901 10.1016/s0195-668x(02)00419-0

[2] Wang, J. , Gu, C. , Gao, M. , Yu, W. & Yu, Y. Preoperative Statin Therapy and Renal Outcomes After Cardiac Surgery: A Meta‐analysis and Meta‐regression of 59,771 Patients. Can. J. Cardiol. 31, 1051–1060 (2015). 26081692 10.1016/j.cjca.2015.02.034

[3] Xiong, B. *et al* Preoperative statin treatment for the prevention of acute kidney injury in patients undergoing cardiac surgery: A meta‐analysis of randomized controlled trials. Heart Lung Circ. 26, 1200–1207 (2017). 28242291 10.1016/j.hlc.2016.11.024

[4] Li, H. , Wang, C. , Liu, C. , Li, R. , Zou, M. & Cheng, G. Efficacy of short‐term statin treatment for the prevention of contrast‐induced acute kidney injury in patients undergoing coronary angiography/percutaneous coronary intervention: A meta‐analysis of 21 randomized controlled trials. Am. J. Cardiovasc. Drugs 16, 201–219 (2016). 26899537 10.1007/s40256-016-0164-5

[5] McWilliam, S.J. , Antoine, D.J. , Smyth, R.L. & Pirmohamed, M. Aminoglycoside‐induced nephrotoxicity in children. Pediatr. Nephrol., 1–11 (2016). 10.1007/s00467-016-3533-z PMC5624973 27848094

[6] Schmitz, C. *et al* Megalin deficiency offers protection from renal aminoglycoside accumulation. J. Biol. Chem. 277, 618–622 (2002). 11700326 10.1074/jbc.M109959200

[7] Sidaway, J.E. *et al* Inhibitors of 3‐hydroxy‐3‐methylglutaryl‐CoA reductase reduce receptor‐mediated endocytosis in opossum kidney cells. J. Am. Soc. Nephrol. 15, 2258–2265 (2004). 15339975 10.1097/01.ASN.0000138236.82706.EE

[8] Antoine, D.J. , Srivastava, A. , Pirmohamed, M. & Park, B.K. Statins inhibit aminoglycoside accumulation and cytotoxicity to renal proximal tubule cells. Biochem. Pharmacol. 79, 647–654 (2010). 19782050 10.1016/j.bcp.2009.09.021

[9] Verhulst, A. , Sayer, R. , De Broe, M.E. , D'Haese, P.C. & Brown, C.D. Human proximal tubular epithelium actively secretes but does not retain rosuvastatin. Mol. Pharmacol. 74, 1084–1091 (2008). 18612079 10.1124/mol.108.047647

[10] Agarwal, R. Statin induced proteinuria: Renal injury or renoprotection? J. Am. Soc. Nephrol. 15, 2502–2503 (2004). 15340001 10.1097/01.ASN.0000143720.71748.79

